# “NÃO É PRIMÁRIA”: a Portuguese version of the SNNOOP10 mnemonic

**DOI:** 10.1055/s-0045-1813240

**Published:** 2025-12-08

**Authors:** Davi Coutinho Marcelino Guerra Leone, Antonio Fernando Soares Menezes Segundo, Alex T. Meira

**Affiliations:** 1 Universidade Federal da Paraíba, Centro de Ciências Médicas, Departamento de Medicina Interna, Serviço de Neurologia, João Pessoa PB, Brazil.; 2 Afya Faculdade de Ciências Médicas da Paraíba, João Pessoa PB, Brazil.

**Keywords:** Headache, Neurologic Examination, Secondary Headache Disorders, Neuroimaging

## Abstract

**Background:**

Secondary headaches arise from underlying medical conditions and are associated with significant morbidity and mortality. Their diagnosis relies primarily on a comprehensive clinical history and a thorough physical examination, both aimed at identifying key warning signs. The SNNOOP10 mnemonic is a widely-recognized tool used to screen for potential secondary causes of headache. It consists of a structured checklist of 15 red flags, with validated sensitivity, specificity, and predictive values. However, the original English version may present challenges for healthcare providers who are non-native English speakers.

**Objective:**

To introduce a culturally-adapted version of the SNNOOP10 mnemonic in Portuguese: “NÃO É PRIMÁRIA” (“IT IS NOT PRIMARY”).

**Methods:**

The authors reorganized the SNNOOP10 red flags into “NÃO É PRIMÁRIA” by semantically grouping related items, assigning each letter to a corresponding clinical element and matching the original red flags into each letter of the new acronym. A comparative table and image were developed to ensure clarity.

**Results:**

The new mnemonic covers all reorganized items of the SNNOOP10 adapted for Portuguese speakers.

**Conclusion:**

“NÃO É PRIMÁRIA” is a practical mnemonic that adapts THE SNNOOP10 for Portuguese-speaking settings, based on clinical experience. It requires formal validation, and future studies should assess its diagnostic accuracy and applicability.

## INTRODUCTION

Secondary headaches result from underlying pathologies and are associated with significant morbidity and mortality. Early and accurate diagnosis is essential and primarily based on a thorough clinical history and detailed physical examination, both of which aim to identify red flags indicative of a secondary etiology. 1 A cross-sectional study conducted in Brazil, 2 based on the criteria stablished on the International Classification of Headache Disorders, second edition (ICHD-2), identified medication overuse as the most prevalent cause of secondary headache, accounting for 16.6% of the cases. Similarly, a Brazilian multicenter, longitudinal, and observational study 3 reported a prevalence of 14.7% of secondary headaches, with medication overuse again being the most common cause (7.1%).

The third edition of the ICHD (ICHD-3) updated the diagnostic criteria for secondary headaches, enabling clinicians to diagnose secondary headaches during the first patient encounter ( Table 1 ). This advancement is based on the understanding that causal relationships can be established before the resolution of the underlying pathology. 4 Recognizing secondary headaches in outpatient and emergency care settings is critical, as these presentations often reflect serious underlying conditions that may require urgent intervention. 4 5

**Table 1:** General diagnostic criteria for the secondary headaches of the ICHD-3

A. Any headache fulfilling criterion C	
Another disorder scientifically documented to be able to cause headache has been diagnosed. Evidence of causation demonstrated by at least two of the following:	1. Headache has developed in temporal relation to the onset of the presumed causative disorder.
	2. Either or both of the following:
	– Headache has significantly worsened in parallel with worsening of the presumed causative disorder; and
	– Headache has significantly improved in parallel with improvement of the presumed causative disorder.
	3. Headache has characteristics typical of the causative disorder.
	4. There is other evidence of causation.
C. Not better accounted for by another ICHD-3 diagnosis.

Abbreviation: ICHD-3, International Classification of Headache Disorders, third edition.

Note: The new ICHD-3 criteria enable clinicians to diagnose secondary headaches in the first patient encounter.

The SNNOOP10 (systemic symptoms including fever, neoplasm in history, neurologic deficit or dysfunction, onset of headache is sudden or abrupt, older age and 10 features beginning with P) mnemonic is a well-established tool used by clinicians to screen for potential secondary causes of headache. It is a systematic checklist comprising 15 red flags with validated sensitivity, specificity, and predictive values. 6 However, the use of the original English version may be challenging for healthcare providers who do not speak English or are non-native English speakers.

To address this limitation, the present study proposes a culturally-adapted version of the SNNOOP10 mnemonic in Portuguese: “NÃO É PRIMÁRIA” (“IT IS NOT PRIMARY”). This adaptation aims to enhance accessibility, promote widespread use, and improve clinical screening in Portuguese-speaking countries.

## METHODS

The present study was conducted with the aim of developing a Portuguese mnemonic to facilitate the memorization of the components of the SNOOP10, which is commonly used in the evaluation of secondary headache red flags. The process began with the conceptualization of a new phrase in Portuguese that would be intuitive and easy to remember for native speakers. The selected phrase, “NÃO É PRIMÁRIA”, was designed to reflect the idea of a non-primary headache, thus inherently suggesting the possibility of a secondary cause.

**Figure 1:**
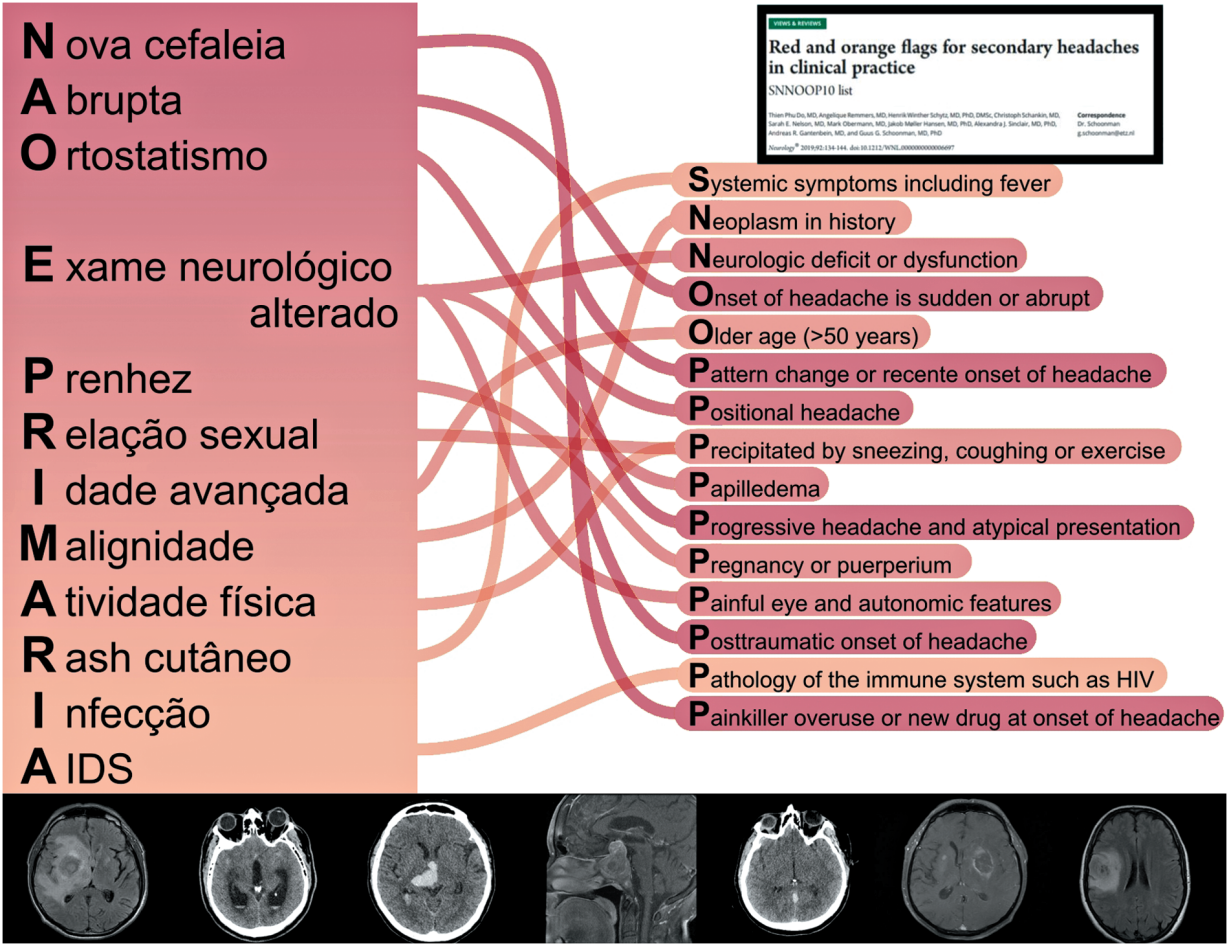
Notes: On the left side, “NÃO É PRIMÁRIA” triggers linked to their SNNOOP10 red flags, on the right side. Below, computed tomography (CT) and magnetic resonance imaging (MRI) scans of examples of secondary etiologies. Visual correlation of “NÃO É PRIMÁRIA” and the SNNOOP10 mnemonic.

To develop the new mnemonic, the authors first grouped the components of the original SNOOP10 acronym based on semantic similarity and thematic proximity. These groupings helped guide the assignment of each element to a corresponding letter in the new acronym. After this initial distribution, each letter of “NÃO É PRIMÁRIA” was assigned a specific clinical item that reflected a component of the SNOOP10. A comparative table ( Table 2 ) was then created to ensure that all original elements were represented accurately in the new format. Finally, a figure ( Figure 1 ) was designed to clearly illustrate the correspondence between the original English acronym and the newly-developed Portuguese version, enhancing clarity and usability for clinical and educational purposes.

**Table 2:** NÃO É PRIMÁRIA versus SNNOOP10

NÃO É PRIMÁRIA	SNNOOP10	Possible etiologies
***N** ova cefaleia *	Pattern change or recent onset of headache; progressive headache and atypical presentation; posttraumatic onset of headache; and painkiller overuse or new drug at onset of headache	Intracranial metastasis; intracranial lesions; venous thrombosis; recently-introduced drug; and painkiller overuse
***A** brupta *	Onset of headache is sudden or abrupt	Subarachnoid hemorrhage; RCVS; sentinel headache; arterial dissection; CVT; intracranial hemorrhage; ischemic stroke; spontaneous intracranial hypotension; and intracranial infections
***O** rtostatismo *	Positional headache	CSF leak
***E** xame neurológico alterado *	Neurologic deficit or dysfunction (including decreased consciousness); papilledema; and painful eye and autonomic features	Intracranial hemorrhage; ischemic strokes; CSF lymphocytosis syndrome; and idiopathic intracranial hypertension
***P** renhez *	Pregnancy or puerperium	Hypertensive disorders; reversible posterior leukoencephalopathy; RCVS; and acute arterial hypertension
***R** elação sexual *	Precipitated by sneezing, coughing or exercise	Atraumatic subarachnoid hemorrhage; basilar artery dissection; RCVS; and Chiari malformation
***I** dade avançada *	Older age (> 50 years)	Stroke; intracranial lesions; and temporal arteritis
***M** alignidade *	Neoplasm history	Brain tumors
***A** tividade física *	Precipitated by sneezing, coughing or exercise	Atraumatic subarachnoid hemorrhage; basilar artery dissection; RCVS; Chiari malformation
**R** ash *cutâneo*	Systemic symptoms including fever	Bacterial or viral meningitis; encephalitis; and neurotuberculosis
**I** nfecção **AIDS** /HIV	Pathology of the immune system such as HIV	CNS infections; cerebrovascular events; epilepsy; and neurodegenerative diseases

Abbreviations: CNS, central nervous system; CSF, cerebrospinal fluid; CVT, cerebral venous thrombosis; RCVS, reversible cerebral vasoconstriction syndrome; SNNOOP10.

Note: The table shows the SNNOOP10 red flags in each letter of the “NÃO É PRIMÁRIA” mnemonic and the possible etiologies that could be associated.

### Não é primária

“NÃO É PRIMÁRIA” is the Portuguese translation of “IT IS NOT PRIMARY.” Each letter refers to a word or combination of words, composing an easy-to-remember and natural mnemonic. The SNNOOP10 topics were redistributed into new divisions; thus, each letter may contain one or more secondary headache red flags, or a single topic may have been divided into two separate items.

Some SNNOOP10 red flags were easily accommodated into the Portuguese mnemonic as their direct translations or near-synonyms, such as “onset of headache is sudden or abrupt,” “positional headache,” “pregnancy and puerperium,” “older age,” and “neoplasm history.” Other red flags were adjusted primarily for pedagogic purposes, using terms that, while not exact matches, effectively evoke the intended situation ( Figure 1 ).

The Portuguese adaption was initially developed by the authors for personal use, grounded in their clinical experience. However, understanding the epidemiological burden and the distress of healthcare providers, the authors conducted a literature review to support the final version of the mnemonic. Each following topic reveals the rationale behind the authors' choice and the possible etiologies behind each red flag ( Table 2 ).

### *Nova cefaleia* (“new headache”)

“Pattern change or recent onset of headache,” “progressive headache and atypical presentation,” “posttraumatic onset of headache,” and “painkiller overuse or new drug at onset of headache” are items from SNNOOP10 mnemonic included in this topic. “Nova cefaleia” means “new headache,” the rationale for grouping this red flags together is that they all share a defining feature - the presence of something new, such as a recent onset, a change in characteristics, or the development of new related habits.

A newly-developed headache or a pattern change may be the only signs of a malignant etiology, such as intracranial metastasis, intracranial lesions, 7 and cerebral venous thrombosis (CVT). 8 A correct diagnosis is often delayed in these cases. Progressive or atypical presentations may be the only evidence of an underlying pathology. Patients with CVT often present with headaches with a progressive course. In one study, 9 two-thirds of chronic headache patients with relevant magnetic resonance imaging (MRI)findings did not meet the criteria for primary headache conditions.

Posttraumatic headache varies depending on sociodemographics. If the headache has a direct correlation with the trauma, a red flag should be raised; if its duration extends, it is an orange flag. Headache due to medication overuse is the most common secondary headache disorder, and its prevalence in Brazil is consistent with global trends. 3 10 Although this condition reduces quality of life and leads to significant disability, it is treatable, but often under-recognized. Treatment is effective, leading to minimal headache burden and lower levels of medication consumption. 11 Furthermore, headaches may be a side effect of a recently-introduced drug. The phenotype can mimic the primary-disorder headaches. One should investigate the exact history to pinpoint the start of medication intake and establish a chronological correlation.

### *Abrupta* (“abrupt”)

“Abrupta” means “abrupt.” The authors included the “onset of headache is sudden or abrupt” red flag in this topic. Thunderclap headache (TCH) is a high-intensity headache that reaches its peak in less than 1 minute, and there are more than 100 causes published in the literature. Subarachnoid hemorrhage (SAH) is the most prevalent etiology. Other causes that should be considered are reversible cerebral vasoconstriction syndrome (RCVS), sentinel headache, arterial dissection, CVT, intracranial hemorrhage, ischemic stroke, spontaneous intracranial hypotension, and intracranial infections. 12 13 The majority are potentially fatal etiologies that require prompt diagnosis and treatment.

In a multicenter cohort study, 14 SAH was identified in 6.2% of the patients with TCH with no neurological signs. Often unrecognized, RCVS may be as common as SAH in the emergency department, once up to 94% of the cases of RCVS presented as TCH according to a systematic review. 15 Sudden and severe headaches not explained by other factors, particularly coital headache, may be indicative of RCVS. 16 17 When detected early, TCH is highly associated with secondary etiologies with good prognosis; thus, physicians must consider it a red flag.

### *Ortostatismo* (“orthostatism”)

We included the red flag “positional headache” in this topic. Positional headache is defined as a headache when assuming an upright position, resolving quickly after lying horizontally. Orthostatic headache is the most common symptom of spontaneous intracranial hypotension. The most prevalent cause is a cerebrospinal fluid (CSF) leak through a tear or hole in the dura at the level of the spine. There are many syndromes associated with this disorder, and normal lumbar puncture and neurosurgical procedures are also frequent and benign etiologies. Spine imaging is essential to discern the probable causes and to locate the CSF leak, which may be from a ventral dural leak, a spine nerve root diverticulum, or a direct CSF venous fistula. Increased awareness of spontaneous intracranial hypotension and correct indication of imaging exams are fundamental for the correct treatment. 18

The occurrence of headaches when assuming different positions besides the upright posture may also indicate secondary etiologies. Two patients were described 19 as having the onset of headache when adopting lateral decubitus orientation. The symptom was caused by middle cerebral artery dissection, confirmed using high-resolution MRI. The headache exacerbated when lying down in lateral decubitus ipsilaterally to the artery dissection. 19 Thus, positional headache is a red flag, given that it may be a frequent symptom of spontaneous intracranial hypotension and may even indicate the laterality of an artery dissection.

### *Exame neurológico alterado* (“abnormal neurological exam”)

In this topic, we included the red flags “neurological deficit or dysfunction,” “papilledema,” and “painful eye and autonomic features.” The rationale behind this topic is based on clinical signs that require a detailed neurological examination and anamnesis to be found.

Neurological deficits are known to be signs of secondary headaches. Intracranial hemorrhage and ischemic strokes are the most common causes. Headache occurred in 16 to 65% of patients with transient ischemic attacks, 20 and, in a prospective study 21 of 240 patients with stroke, they were observed in 64.5% of the cases of hemorrhagic stroke and in 32% of the cases of ischemic stroke. The manifestations of CSF lymphocytosis syndrome also include unilateral severe throbbing episodic headache associated with sensory deficit (60%) and motor deficits (54.8%). 22

Idiopathic intracranial hypertension is a potentially serious condition that requires timely diagnosis and management to prevent permanent visual impairment. The two most frequent symptoms are papilledema and chronic headache, typically described as diffuse head pain radiating to the posterior neck. Additional manifestations may include visual deterioration, cranial nerve palsies, and cognitive deficits. 23 The presence of papilledema warrants investigation into the underlying cause of increased intracranial pressure, typically involving radiological imaging. 24

A relevant proportion of secondary trigeminal autonomic cephalalgias (TACs) may present all diagnostic criteria for primary TAC. Hence, it is suggested that neuroimaging should be performed in all TAC patients. The etiologies vary, ranging from vascular to structural disorders. 25 A report 26 described a 44-year-old patient with episodic right-sided headaches for 1 month, 1 to 3 times a day, with narrowing of the palpebral fissure and lacrimation. Digital subtraction angiography revealed a right indirect carotid cavernous fistula, with complete relief of the headache after endovascular embolization. 26 Furthermore, Pelikan et al. 27 reported a case of a 42-year-old female patient with sudden-onset, left-sided headache with ipsilateral autonomic symptoms, who also developed numbness on the left side of the lips and ataxia. An MRI scan revealed multiple demyelinating plaques. Her headaches resolved after dimethyl fumarate treatment. 27

### *Prenhez* (“pregnancy”)

Here, we included the red flag “pregnancy and puerperium.” Pregnancy increases the risk for many causes of headache, including pathologic vascular conditions, due to physiologic changes and the greater burden of interventions. A 2015 retrospective study 28 found a rate of 35% of secondary headaches among 140 pregnant women with acute headache. Hypertensive disorders, reversible posterior leukoencephalopathy, RCVS, and acute arterial hypertension were the most prevalent causes. 28 Other probable causes are CVT, ischemic stroke, SAH, and arterial dissection. 29

The clinical features of secondary headaches in pregnancy and puerperium may be identical to those of primary headaches. For instance, migraine alone is an independent risk factor, associated with a 1.42-fold increase in the risk of gestational hypertension (odds ratio: 2.3; 95%CI: 2.1–2.5). 30 The differential diagnosis can be challenging and may prompt questions about the need for screening for secondary causes in pregnant women presenting with classic symptoms of primary headaches.

### *Relação sexual e atividade física* (“sex and physical activity”)

The SNNOOP10 red flag “precipitated by sneezing, coughing or exercise” was divided into two topics: sexual intercourse and physical activity headaches. The reasoning is to aggregate conditions that raise internal pressure, in which the patient performs some kind of effort. We structured it this way simply for pedagogical reasons and to suit the mnemonic.

A retrospective study 31 in an emergency department analyzed 129 radiological exams performed because of sexual intercourse complaints. The most common sexual intercourse-associated pathology was headache attributed to cerebrovascular insult, with a higher prevalence in men and atraumatic SAH being the most frequent etiology. 31 Furthermore, there are reports 32 33 of basilar artery dissection and RCVS presenting with thunderclap headache after orgasm.

Half of the patients with hypertension in the emergency department report headache. The hypertension headache phenotype is usually bilateral or diffuse with pulsating quality and tends to worsen with physical activity. 34

Cough headache is associated with Chiari malformation and other posterior fossa lesions. 35 In a study with 19 type-1 Chiari malformation patients, 35 10 complained of headache. The headache type was analyzed, and the authors found 6/10 cases associated with coughing, 6/10, with sexual activity, 5/10, with effort and 1/10 cases associated with sneezing.

Thus, sexual intercourse, physical activity, and Valsalva-related conditions are red flags for secondary headache.

### *Idade avançada* (“older age”)

Headache starting in older patients is categorized as a red flag, because of the higher frequency of secondary causes of alarming etiology in this age group, such as stroke, intracranial lesions and temporal arteritis.

Older patients are 12 times more likely to have serious underlying causes and atypical symptomatic presentation compared with younger adults. Treatment could be limited in older patients, comorbid medical conditions are common and affect some therapy results and side-effects. 36

### *Malignidade* (“malignancy”)

Here, we included the red flag “neoplasm in history.” The term “ *malignidade* ” is a direct translation of “malignancy,” and it serves as a reminder to identify patients with an active neoplasm or history of it.

Active cancer or previous neoplasia, particularly with brain-tropic tumors, combined with red flags such as new or atypical headache, markedly raises the suspicion of intracerebral metastases. 37 Headache occurs in 32.2 to 71% of the patients with brain tumors, regardless of tumor type, with both primary and metastatic lesions presenting similar likelihoods of causing headache. It is important to keep in mind that brain tumor-related headaches rarely present in isolation. 38 In a prospective study, the authors 39 identified four independent predictors: pulsating quality and moderate-to-severe intensity, emesis, gait instability, and extensor plantar response. Thus, every oncology patient presenting with headache should undergo a more in-depth examination with imaging exams.

### Rash *cutâneo* (“skin rash”)

In this topic, we included the SNNOOP10 red flag “systemic symptoms including fever.” Skin rash is used pedagogically within the mnemonic to prompt the reader to recall that systemic symptoms—particularly fever—constitute a red flag in patients presenting with headache.

Fever is one of the most frequent complaints in the emergency department and in outpatient visits, but, in a study, 40 only 1 out of 213 non-surgical, febrile patients had fever due to neurological infection. The association of fever and headache heightens the clinical sensitivity to secondary etiologies, prompting the inclusion of neurological infections in the differential diagnosis. Van de Beek et al. 41 found that, among 696 cases of bacterial meningitis, 87% had headache and 77%, fever. Furthermore, in a study 42 with 113 encephalitis cases, 79% had headache and 84%, fever. In Brazil, in a tertiary hospital, 43 neurotuberculosis was the most frequent bacterial infection found.

Fever alone is considered an orange flag, as it may result from a wide range of infectious or inflammatory conditions. Its association with other significant symptoms, however, elevates it to a red flag. Thus, clinicians must consider all range of systemic symptoms, not limiting themselves only to rash.

### *Infecção* /AIDS (“Infection/AIDS”)

This topic addresses the “pathology of the immune system such as HIV” red flag as originally outlined in the SNNOOP10 criteria. “Infection” and “AIDS” were used to fit the mnemonic and serve as a trigger to recall opportunistic infections and HIV infection respectively. Both are pathologies of the immune system.

The burden of headache in HIV-positive patients is high. The 1-year prevalence of headache in these patients was of 80.3% in a sub-Saharan study that included 498 individuals. 44 In a study, 45 HIV-positive men aged between 40 and 49 years receiving highly-active antiretroviral therapy have a greater incidence of neurologic diagnoses than HIV-negative men (11.6 versus 2.0 respectively; *p* < 0.001). Infection by HIV is associated with a broad spectrum of neurological diagnoses, such as central nervous system infections, cerebrovascular events, seizure disorders, and neurodegenerative diseases. 45 Clinicians could encounter opportunistic infections—such as neurotoxoplasmosis, herpes zoster, neurotuberculosis, and cryptococcal meningitis—in HIV-positive patients who are either untreated, severely immunosuppressed, or experiencing antiretroviral therapy failure. 44

Pathologies of the immune system, especially HIV, are a red flag due to the high risk of other neurological pathologies associated with this diagnosis.

In conclusion, “NÃO É PRIMÁRIA” is a helpful, easy-to-use and evidence-based tool for neurologists and primary care and emergency doctors. It refines the use of the SNNOOP10 in a Portuguese-speaking country, increasing the odds of detecting a secondary cause of headache. The current study draws on the clinical experience of three authors and has not undergone validation by a panel of neurologists or other subject-matter experts. Future studies may address the validation of each red flag herein presented.

Future research may include retrospective analyses to assess rare secondary headache etiologies and their associated risk factors, as well as prospective studies aimed to validate the proposed red flags. Additionally, comparative studies between services that implement the mnemonic and those that do not could evaluate the impact on diagnostic accuracy for secondary headaches and their sensitivity, specificity and predictive values.
